# Reply to: “Steller’s sea cow uncertain history illustrates importance of ecological context when interpreting demographic histories from genomes”

**DOI:** 10.1038/s41467-022-31382-5

**Published:** 2022-06-28

**Authors:** Fedor S. Sharko, Sergey M. Rastorguev, Alexei N. Tikhonov, Artem V. Nedoluzhko

**Affiliations:** 1 Research Center of Biotechnology of the Russian Academy of Sciences, Leninsky prospect 33/2, 119071 Moscow, Russia; 2 National Research Center “Kurchatov Institute”, 1st Akademika Kurchatova Square, 123182 Moscow, Russia; 3 Limited liability company ELGENE, Malaya Kalitnikovskaya 16, 109029 Moscow, Russia; 4 Zoological Institute Russian Academy of Sciences, Universitetskaya nab., 1, 199034 Saint-Petersburg, Russia; 5 Institute of Applied Ecology of the North, North-Eastern Federal University, Lenina 43, 677980 Yakutsk, Russia; 6 European University at St. Petersburg, Shpalernaya Ulitsa 1, 191187 St. Petersburg, Russia

**Keywords:** Evolutionary genetics, Conservation biology, Evolutionary biology

**replying to** A. A. Campos et al. *Nature Communications* 10.1038/s41467-022-31381-6 (2022)

A Matters Arising article raised concerns about the interpretation of our findings reported in our recent publication on the Steller’s sea cow (*Hydrodamalis gigas*) nuclear genome^1^. After careful consideration of the criticism, we maintain our main conclusion that this marine mammal started to become extinct at a period significantly preceding the arrival the first Paleolithic humans in the Bering Sea region. This conclusion is supported not only by genomic analysis but also by the small number of Steller’s sea cow bones that were excavated in prehistorical archeological sites along the North Pacific coast line^2–5^.

We suppose that extinct the Steller’s sea cow could migrate significant distances along the coastline and between islands (including Aleutian and Commander Islands) in the same way as the extant sirenian species: the dugong (*Dugong dugon*)^6^ and the West Indian manatee (*Trichechus manatus*)^7^. Although Steller’s sea cow was likely unable to swim across large distances it could conquer new island habitats due to the westward Alaskan current that flows along the south side of the Alaska Peninsula and the Aleutian Islands^8^ and enters the Bering Sea through several Aleutian passes, and then forms the Aleutian North Slope current that flows into the East Kamchatka current (Fig. 1)^9^. Moreover, this sea current configuration has existed since at least the Middle Pleistocene despite glaciations and the Bering Sea level drop^9^.

**Fig. 1:**
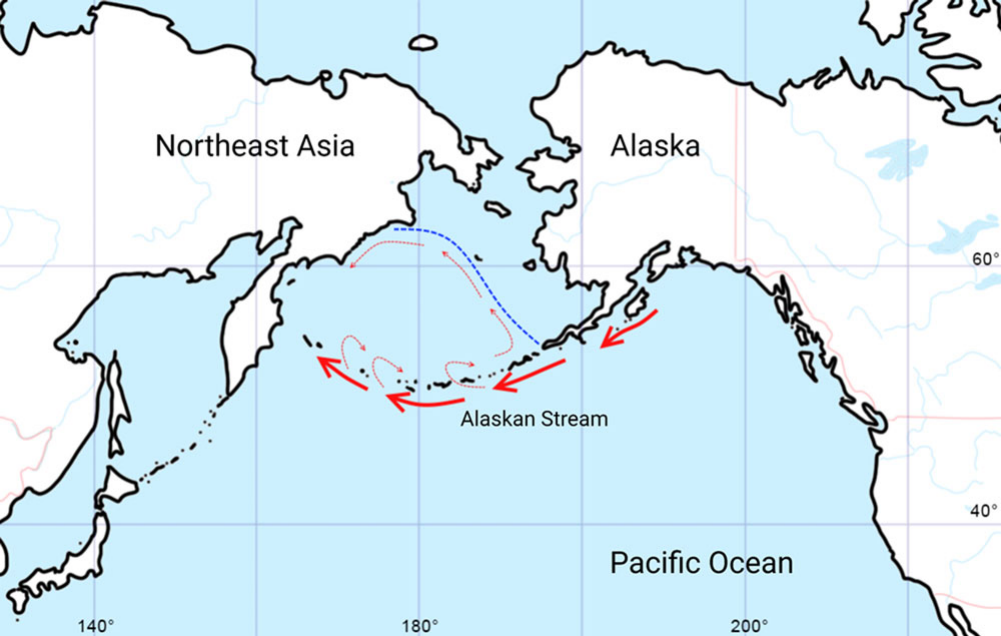
Simplified representation of the sea current configuration in North Pacific region. The blue dashed line schematically represents the Last Glacial Maximum coastline and the red arrows show the Alaskan Stream, which enters the Bering Sea, then forms the Aleutian North Slope (marked by red dashed arrows) current which flows into the East Kamchatka current (modified from Detlef et al.^9^).

Thus, the Alaskan stream could allow in a constant westward gene flow among the *H. gigas* populations of Alaska and the Aleutian and the Commander Islands, which, at the current time, is the case for northern fur seals’ (*Callorhinus ursinus*) early migrations. Fur seal pups born on St. Paul Island usually migrate westward without being able to dive deeply, feed or navigate^10^.

Sea otter hunting (*Enhydra lutris*) was one of the possible indirect reasons for the extinction of the last *H. gigas* population on the Commander Islands. Some think that fur traders used Steller’s sea cows as a source of meat and fat^2^; others propose that overhunting of the sea otter influenced local ecological food chains and led to the loss of kelp forests and to the parallel extinction of the last *H. gigas* population, which lost its feeding source^11^. Nevertheless, sea otters (strong swimmers and divers) survived as a species after this “fur trade fever” with the repeated loss of genetic diversity along the North Pacific Ocean. Moreover, the gene flow between the islands and the mainland populations still remains^12^. It is also suggested that the extinction of Steller’s sea cow in the North Pacific region had a significant impact on kelp forests and ecosystem dynamics in general^13^.

Modern data on the genetic diversity of another, and genetically closest, sirenian species, the dugong, showed low genetic diversity levels in the Indian and the Pacific Ocean populations and detected geographic structuring among them. Several mitochondrial lineages of this species have been described^14^. At the same time, these obtained mitochondrial DNA data present the migration possibilities for this species (predominantly by males) along the coastline of Africa, Asia, Australia, and possibly between parts of the Indonesian archipelago^14,15^.

In our study that described the first nuclear genome of *H. gigas*^1^, we did not propose a panmictic character of the population structure for the Steller’s sea cow in its distribution limits. However, it seems that gene flow between the Commander and Aleutian Islands *H. gigas* was possible until Last Glacial Maximum, which took place from 26.5 ka to 19 to 20 ka^16^. We suppose that much earlier glaciations and periods in between in this region could limit as well as enhance the gene flow of animal and plant species in the North Pacific Rim. Moreover, kelp forests have grown along the Aleutian–Commander Islands chain and further towards the Kuril Islands and Japan continuously.

In conclusion, we suggest that precise geological data on the Bering Sea level during the Pleistocene and additional *H. gigas* genomic datasets from individuals representing different periods and geographical localities will be necessary to determine the causes for the extinction of the Steller’s sea cow in the North Pacific region.

## Reporting summary

Further information on research design is available in the Nature Research Reporting Summary linked to this article.

## Supplementary information

Reporting Summary

## Data Availability

All literature sources used in this article are available in the References list.
