# Supplementary material for: Reply to: “Steller’s sea cow uncertain history illustrates importance of ecological context when interpreting demographic histories from genomes”
Source: Nat Commun. 2022 Jun 28;13:3672. doi: 10.1038/s41467-022-31382-5 (PMC9240042; doi:10.1038/s41467-022-31382-5)
Supplement: Supplementary file 1 — Reporting Summary [file 41467_2022_31382_MOESM1_ESM.pdf]

nature portfolio

Corresponding author(s): Artem Nedoluzhko

Last updated by author(s): May 17, 2022

Reporting Summary

Nature Portfolio wishes to improve the reproducibility of the work that we publish. This form provides structure for consistency and transparency in reporting. For further information on Nature Portfolio policies, see our Editorial Policies and the Editorial Policy Checklist.

Statistics

For all statistical analyses, confirm that the following items are present in the figure legend, table legend, main text, or Methods section.

n/a	Confirmed	
☒	☐	The exact sample size (*n*) for each experimental group/condition, given as a discrete number and unit of measurement
☒	☐	A statement on whether measurements were taken from distinct samples or whether the same sample was measured repeatedly
☒	☐	The statistical test(s) used AND whether they are one- or two-sided<br>*Only common tests should be described solely by name; describe more complex techniques in the Methods section.*
☒	☐	A description of all covariates tested
☒	☐	A description of any assumptions or corrections, such as tests of normality and adjustment for multiple comparisons
☒	☐	A full description of the statistical parameters including central tendency (e.g. means) or other basic estimates (e.g. regression coefficient) AND variation (e.g. standard deviation) or associated estimates of uncertainty (e.g. confidence intervals)
☒	☐	For null hypothesis testing, the test statistic (e.g. *F*, *t*, *r*) with confidence intervals, effect sizes, degrees of freedom and *P* value noted<br>*Give P values as exact values whenever suitable.*
☒	☐	For Bayesian analysis, information on the choice of priors and Markov chain Monte Carlo settings
☒	☐	For hierarchical and complex designs, identification of the appropriate level for tests and full reporting of outcomes
☒	☐	Estimates of effect sizes (e.g. Cohen's *d*, Pearson's *r*), indicating how they were calculated

Our web collection on statistics for biologists contains articles on many of the points above.

Software and code

Policy information about availability of computer code

Data collection	This study involved no data collection
Data analysis	This study ivnolved only literature analysis

For manuscripts utilizing custom algorithms or software that are central to the research but not yet described in published literature, software must be made available to editors and reviewers. We strongly encourage code deposition in a community repository (e.g. GitHub). See the Nature Portfolio guidelines for submitting code & software for further information.

Data

Policy information about availability of data

All manuscripts must include a data availability statement. This statement should provide the following information, where applicable:

- Accession codes, unique identifiers, or web links for publicly available datasets
- A description of any restrictions on data availability
- For clinical datasets or third party data, please ensure that the statement adheres to our policy

All literature sources used in this article are available in the References list.

Field-specific reporting

Ecological, evolutionary & environmental sciences study design

All studies must disclose on these points even when the disclosure is negative.

Study description	In this study we analyze the migration possibility of the Steller's sea cow specimens from the main part of its range (e.g. Aleutian Islands) to the Commander Islands based on previously published literature sources.
Research sample	In this study we discuss Steller's sea cow specimen (field number of the sample: Ber/07/Yk-1), whose genome has been sequenced and has been published in 2021: Sharko, F.S., Boulygina, E.S., Tsygankova, S.V. et al. Steller's sea cow genome suggests this species began going extinct before the arrival of Paleolithic humans. Nat Commun 12, 2215 (2021). https://doi.org/10.1038/s41467-021-22567-5
Sampling strategy	This study involved no data collection
Data collection	This study involved no data collection
Timing and spatial scale	This study involved no data collection
Data exclusions	This study involved only literature analysis
Reproducibility	This study involved only literature analysis
Randomization	This study involved only literature analysis
Blinding	This study involved only literature analysis

Did the study involve field work? ☐ Yes ☒ No

Reporting for specific materials, systems and methods

We require information from authors about some types of materials, experimental systems and methods used in many studies. Here, indicate whether each material, system or method listed is relevant to your study. If you are not sure if a list item applies to your research, read the appropriate section before selecting a response.

Materials & experimental systems

n/a	Involved in the study	
☒	☐	Antibodies
☒	☐	Eukaryotic cell lines
☒	☐	Palaeontology and archaeology
☒	☐	Animals and other organisms
☒	☐	Human research participants
☒	☐	Clinical data
☒	☐	Dual use research of concern

Methods

n/a	Involved in the study	
☒	☐	ChIP-seq
☒	☐	Flow cytometry
☒	☐	MRI-based neuroimaging
